# The role of stride frequency for walk-to-run transition in humans

**DOI:** 10.1038/s41598-017-01972-1

**Published:** 2017-05-17

**Authors:** Ernst Albin Hansen, Lasse Andreas Risgaard Kristensen, Andreas Møller Nielsen, Michael Voigt, Pascal Madeleine

**Affiliations:** Research Interest Group of Physical Activity and Human Performance, SMI®, Department of Health Science and Technology, Aalborg University, Fredrik Bajers Vej 7D, 9220 Aalborg, Denmark

## Abstract

It remains unclear why humans spontaneously shift from walking to running at a certain point during locomotion at gradually increasing velocity. We show that a calculated walk-to-run transition stride frequency (70.6 ± 3.2 strides min^−1^) agrees with a transition stride frequency (70.8 ± 3.1 strides min^−1^) predicted from the two stride frequencies applied during treadmill walking and running at freely chosen velocities and freely chosen stride frequencies. The agreement is based on Bland and Altman’s statistics. We found no essential mean relative difference between the two transition frequencies, i.e. −0.5% ± 4.2%, as well as limits of agreement of −8.7% and 7.7%. The particular two freely chosen stride frequencies used for prediction are considered behavioural attractors. Gait is predicted to be shifted from walking to running when the stride frequency starts getting closer to the running attractor than to the walking attractor. In particular, previous research has focussed on transition velocity and optimisation theories based on minimisation of, e.g., energy turnover or biomechanical loadings of the legs. Conversely, our data support that the central phenomenon of walk-to-run transition during human locomotion could be influenced by behavioural attractors in the form of stride frequencies spontaneously occurring during behaviourally unrestricted gait conditions of walking and running.

## Introduction

A better understanding of the control of bipedal locomotion can contribute to the development of programs for the enhancement of human function and performance. An intriguing long-standing question which has been investigated by researchers within, for example, neuroscience, physiology, and psychology is, “Why do humans spontaneously shift from walking to running at a certain point during locomotion at gradually increasing velocity?”^1, 2^ The answer to this question remains unclear^3, 4^. A considerable proportion of the research within this field has focussed particularly on the transition velocity^1–6^ as well as on optimisation theories based on minimisation of, for example, rate of energy turnover^5^ or biomechanical loadings of the legs^6^. In addition, computer modelling has been performed to increase our understanding^4, 7^. The latter approach caused Hubel and Usherwood (2013) to suggest that an inverted pendulum gait model has predictive power in regard to gait transitions. Srinivasan and Ruina (2006) used a similar approach and found that at low velocities a computer-based energetically optimization discovered the classic inverted-pendulum walk while a bouncing run occurred at high velocities. At intermediate velocities, a new pendular-running gait type was found.

Bipedal locomotion constitutes a fundamental characteristic of humans. During human walking, imposed increasing velocity initially causes individuals to concomitantly increase the stride frequency and stride length^8^. Furthermore, the gait type shifts from walking to running^9, 10^ at a velocity of approximately 7.6 km h^−1^. This shift or transition occurs despite the fact that walking can still be easily performed at higher velocities. Previous attempts to explain the transition have often focused on the velocity at which the walk-to-run transition occurs. In comparison, a principal novel aspect of the present study is the focus on the stride frequency in relation to the walk-to-run transition. Based on reviews of the literature, some authors have hypothesised that the walk-to-run transition is primarily driven by an intention to minimize the rate of energy turnover^5, 11^. However, this hypothesis has been challenged^12, 13^. The previously reported coincidence between the energetically optimal and the freely chosen stride frequencies in locomotion possibly reflects the evolutionary development rather than deliberate motor control prioritising minimisation of the energy turnover. Others have suggested that the transition is triggered by increased sensed effort due to exaggerated biomechanical loading in the form of increased activation of the tibialis anterior, rectus femoris, and hamstring muscles during the swing phase^3, 14^. Finally, others have advocated that cognition might play a role since the cognitive load has been shown to occupy the attentional resources, which contribute to trigger the transition during human gait^15, 16^. Nevertheless, the body of the existing literature concerning the walk-to-run transition does not provide any clear-cut explanation of the reason for the transition^6^. Knowledge on adaptations to changes in velocity during locomotion is of importance in regard to the basic understanding of the underlying control of locomotion. In this relation, the walk-to-run transition constitutes a central aspect of locomotion.

With regard to generation of locomotion in general as well as to the more specific focus of the present study, two considerations are of particular relevance. Initially, a common understanding of the generation of locomotion^17, 18^ as well as rhythmic movement in general^19^ is that the spinal neural networks, termed central pattern generators (CPGs), play a key role. The CPGs are considered to mediate an organized pattern of motor activity in combination with adequate supraspinal descending and peripheral afferent influences. For reviews on this topic, the reader is referred to previously published articles^18, 20–22^. The existence of CPGs and homologous interneurons has been proven in several vertebrate species (lampreys^23^, mice^24^, cats^25^). In comparison, it has been difficult to directly prove the existence of the CPG function in primates^26^ and humans; although indirect evidence of the existence of functional CPG-like spinal neural networks has been reported in patients with spinal cord injuries^27, 28^ and in infants^29^. A second consideration of the generation of locomotion is that behavioural attractors could play a role^30^. The present and other studies^10, 30^ consider a behavioural attractor as a behavioural mode. More specifically, we considered the stride frequencies during unrestricted walking and running at freely chosen velocity and freely chosen stride frequency to constitute two influential behavioural attractors. Moreover, if these behavioural attractors indeed significantly affect the gait transition from walking to running, the transition could hypothetically be predicted to occur at a point where the applied stride frequency during walking at increasing velocity starts to get closer to the attractor for running than to the attractor for walking. The link between the stride frequencies during walking and running, the motor control, and the two behavioural attractors described above is that to a substantial degree, the latter apparently represents basic frequency outputs from the CPGs.

**Figure 1:**
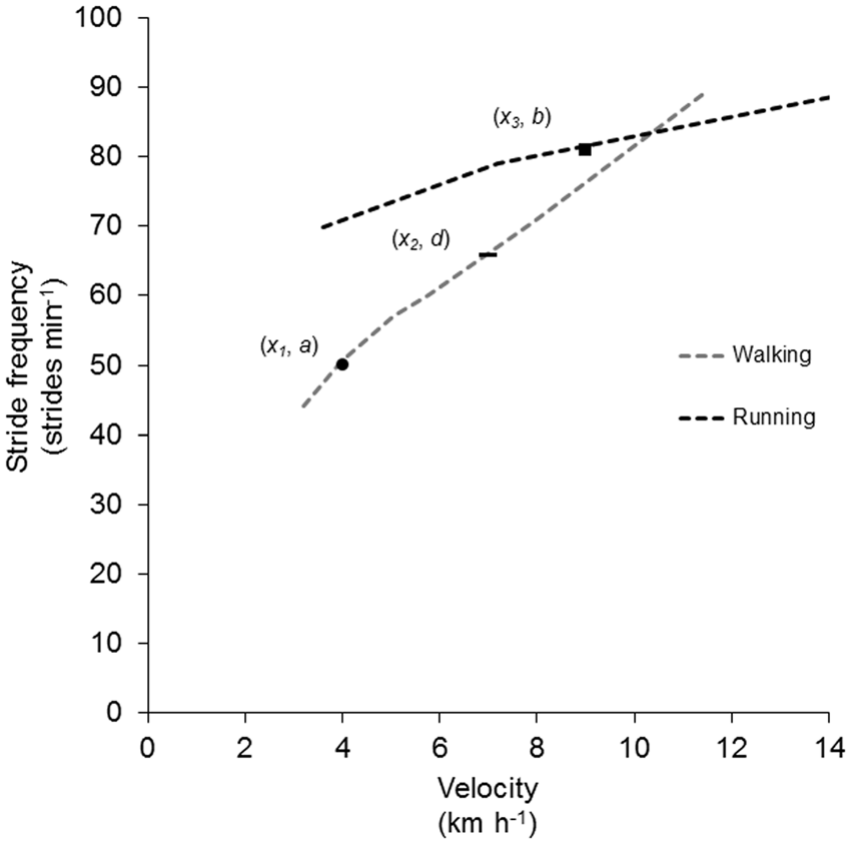
Illustration of the underlying justification for the predicted transition stride frequency in the present study. Broken lines represent the common relationships between freely chosen stride frequencies and velocity during walking and running at ranges of pre-set velocities. The lines were produced using experimentally collected mean data from 10 participants^48^. Hypothetical data points are inserted to represent a single individual during unrestricted gait conditions of walking (black filled circle) and running (black filled square) at freely chosen velocities and freely chosen stride frequencies. The stride frequencies (i.e. *y*-coordinates) applied during these two gait conditions were considered behavioural attractors. The rationale for the predicted transition stride frequency was as follows: When walking at increasing velocity, individuals shift from walking to running when the stride frequency starts getting closer to the attractor “ahead” than to the attractor “behind”. Accordingly, the predicted transition stride frequency (*d*) for the hypothetical individual in this case constitutes the *y*-coordinate of the superimposed data point of the short black horizontal bar.

Figure 1 gives a detailed illustration of the justification and procedure for the prediction of the walk-to-run transition stride frequency. The prediction is based on the combined consideration of the CPG-mediated locomotion and the behavioural attractors as described above. Further, the prediction implies a shift of the walking at increasing velocity into running when the stride frequency gets closer to the running attractor than to the walking attractor. This is reflected by the constant of 0.5 in eq. 1 (see Methods). The prediction includes that walking could easily be continued at higher velocities, beyond the transition velocity at which a shift to running is preferred. The reason for the particular preference is unknown, but may involve an individual’s motor memory and available motor repertoires. Thus, the individual might be well aware of the deviance from an applied stride frequency to the forthcoming behavioural attractor. An inversed protocol starting with fast running followed by a decrease of the velocity often results in a similar transition velocity^2, 14^. However, according to Fig. 1 it is evident that in case of an inversed protocol the stride frequency is still close to the running attractor when the shift from running to walking is made. The reason why the shift is nevertheless made might be that running becomes an awkward and unnatural type of gait at the relatively low velocities just below the transition velocity. Accordingly, an individual may possibly make the shift from running to walking at a point where running becomes awkward and causes the individual to explore the motor repertoire for an alternative type of gait; in this case walking. Other researchers working within the present topic have also discussed that there might be different explanations for the walk-to-run and run-to-walk transition including varied amounts of integrated electromyographic activity of the tibialis anterior muscle^31^.

For comparison with the predicted walk-to-run transition stride frequency, a calculated transition stride frequency was considered. The latter was determined at the transition velocity which is the velocity at which the transition from walking to running occurs^1^. We hypothesised that in spite of the independence of the two different ways of determining the transition stride frequencies, an agreement would be found between the calculated and the predicted walk-to-run transition stride frequencies. To test the hypothesis, we recruited 26 healthy individuals to perform locomotion at a freely chosen stride frequency on a motorized treadmill at pre-set stepwise increasing velocities as well as at freely chosen velocities (see Methods section).

## Results

### Calculated walk-to-run transition stride frequency

**Figure 2:**
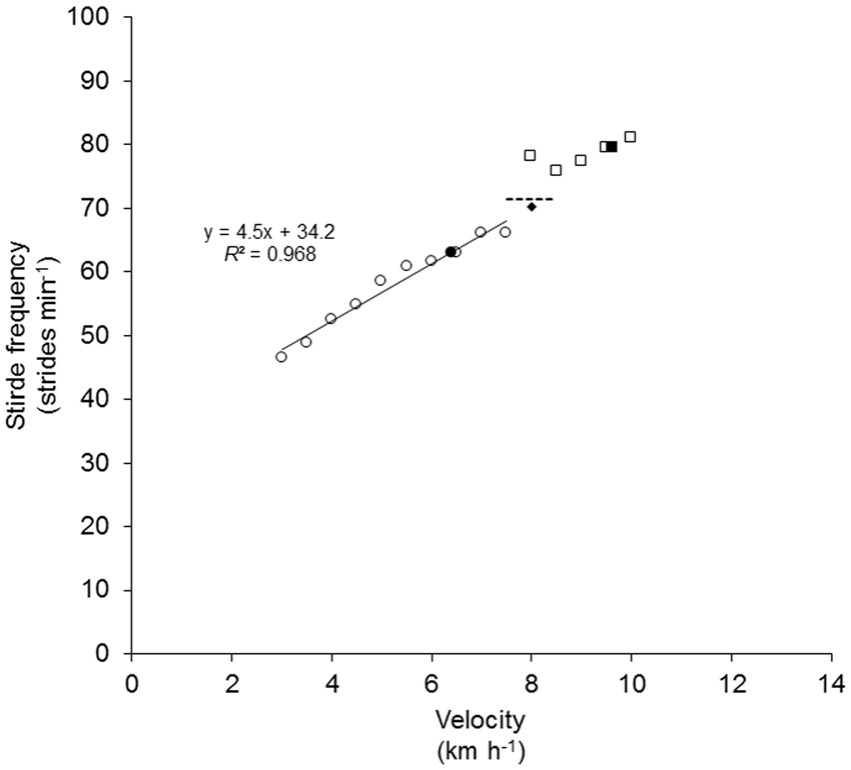
Data example (*n* = 1) of stride frequency (open circles for walking and open squares for running) as a function of velocity. These data were obtained in the part of the present protocol in which increasing velocity was applied. In this particular case, the walk-to-run transition was observed to occur at 8.0 km h^−1^. A linear regression line is superimposed on the data from walking (open circles). The equation for this line was used to determine the calculated transition stride frequency by inserting the relevant walk-to-run transition velocity of 8.0 km h^−1^. The calculated transition stride frequency as a function of the observed walk-to-run transition velocity is indicated by a filled diamond. Included are also the data points (filled circle and square) from the part of the protocol in which the conditions (velocity and stride frequency) of walking and running were freely chosen. The *y*-coordinates of these two data points represent the two behavioural attractors. The predicted transition stride frequency, which was based on the two attractors as outlined in Fig. 1, is indicated by a short horizontal broken line.

The participants showed an increasing stride frequency with increasing velocity during walking at the pre-set stepwise increasing velocities, starting at 3 km h^−1^. Thus, the coefficient of determination (*R* ^*2*^-value, obtained from two-tailed Pearson product-moment correlations) from the correlation between the stride frequency and velocity was 0.976 (*SD* 0.02) as a mean across participants. Figure 2 illustrates a data example. Notably, the within-individual day-to-day relative reliability of freely chosen stride frequencies during locomotion has previously been reported to be almost perfect^32^ with intraclass correlation (*ICC*) values ranging between 0.80 and 0.94^33^. The velocity at which the participants shifted from walking to running was 7.7 (*SD* 0.4) km h^−1^. In comparison, others have previously reported similar walk-to-run transition velocities of 8.05^6^ and 7.56^9^ km h^−1^. For each participant, the calculated transition stride frequency was determined based on the individual walk-to-run transition velocity and the individual equation of the linear regression between velocity and stride frequency during walking. This calculated transition stride frequency amounted to 70.6 (*SD* 3.2) strides min^−1^ as a mean across participants. A similar transition stride frequency of 70.2 has previously been reported^2^.

### Predicted walk-to-run transition stride frequency

Allowing the participants to walk and run in unrestricted gait conditions, i.e. at freely chosen velocities and freely chosen stride frequencies, resulted in a velocity of 5.3 (*SD* 0.8) km h^−1^ and a stride frequency of 58.8 (*SD* 4.2) strides min^−1^ during walking. During running, the corresponding data were 10.5 (*SD* 1.6) km h^−1^ and 82.8 (*SD* 3.5) strides min^−1^, respectively. These two stride frequencies during walking and running were considered behavioural attractors. The predicted walk-to-run transition stride frequency based on these attractors was 70.8 (*SD* 3.1) strides min^−1^.

### Agreement analysis

**Figure 3:**
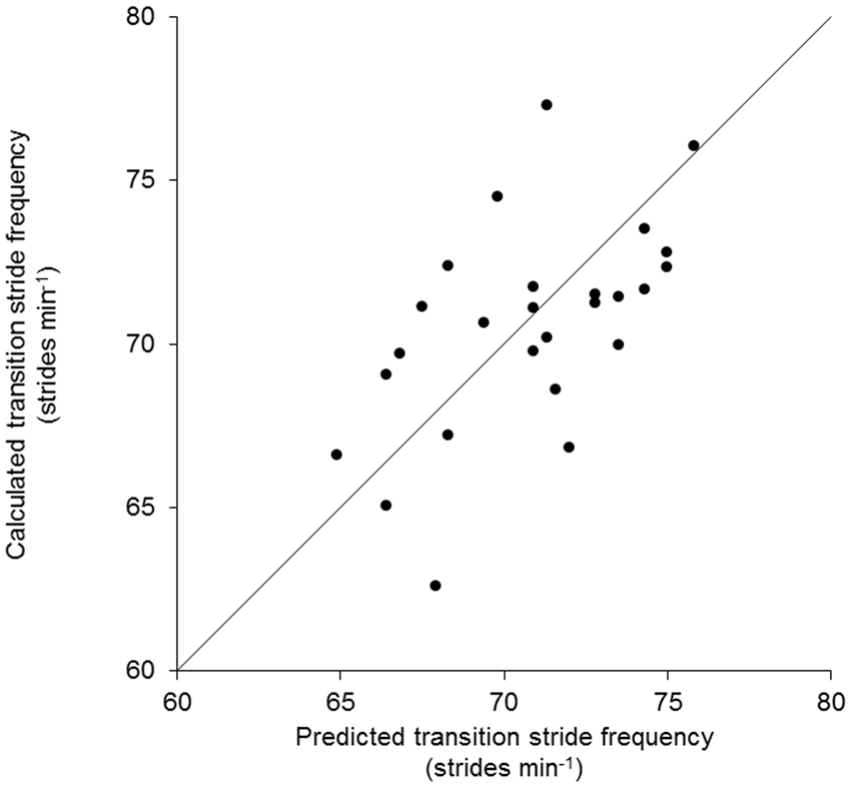
Calculated vs. predicted transition stride frequency. A two-tailed Pearson product-moment correlation was applied for correlation analysis. The coefficient of determination (*R* ^2^) is 0.316 (*P* = 0.003). The line of equality is superimposed (*n* = 26).

**Figure 4:**
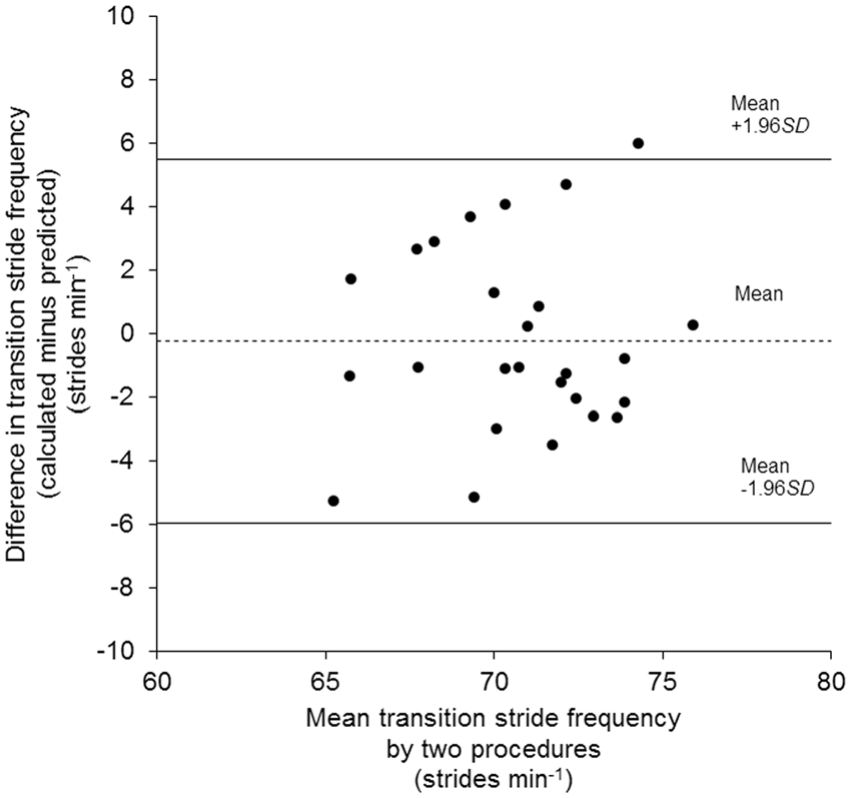
Bland-Altman plot^35^. The difference between the two applied methods for determination of the transition stride frequency (predicted and calculated) is depicted as a function of the mean value by the two methods (*n* = 26). The mean difference of −0.3 strides min^−1^ is illustrated by a broken line while ± 1.96 × *SD* thresholds of 5.5 and −6.0 strides min^−1^ are illustrated by solid lines. A two-tailed Pearson product-moment correlation was applied for correlation analysis. The coefficient of determination (*R* ^2^) is 0.004 (*P* = 0.771).

Firstly, in the analysis of agreement between the calculated and the predicted stride frequencies at walk-to-run transition, we hypothesized that the two transition stride frequencies differed by no more than a mean difference of 2 strides min^−1^ (corresponding to 2.8%). This was done by applying two one-sided tests in SPC Excel Add-in (QIMacros, KnowWare International Inc., Denver, CO, USA). The test showed that the values of the calculated and the predicted walk-to-run transition stride frequencies were equivalent (*P* = 0.025 and 0.006). Notably, a statistical power calculation revealed that with the present number of participants (*n* = 26), a difference of 2 strides min^−1^ (corresponding to 2.8%) in the transition stride frequency between the two applied methods could have been detected considering a power level of 0.80 and a *P*-value of 0.05. Secondly, there was a fair^34^ correlation (*R* = 0.56) between the calculated and predicted transition stride frequencies (Fig. 3). Thirdly, a Bland-Altman plot^35^ was prepared (Fig. 4). The plot revealed no essential mean difference between the two determinations of the walk-to-run transition stride frequency. Further, in 95% of the cases individually calculated values could be expected to be determined by the predicted values and *vice versa* within limits of agreement of ±5.8 strides min^−1^.

To further investigate the role of stride frequency in the walk-to-run transition, we performed additional analyses. Thus, stride frequency was replaced by respectively velocity, stride length, and Froude number (see Methods section) for prediction and calculation of walk-to-run transition as well as for the subsequent analysis of agreement. For velocity, the correlation between the predicted (7.92 (*SD* 0.90) km h^−1^) and calculated transition velocity (7.67 (*SD* 0.45) km h^−1^) resulted in an *R* = 0.48 (*P* = 0.013)(see Supplementary Fig. S1). The difference between the two transition velocities was −3.2% (*SD* 10.3%). A Bland-Altman plot had limits of agreement of −23.3% and 16.9% and a regression equation of y = −0.87x + 6.6, with an *R* = −0.65 (*P* < 0.001)(see Supplementary Fig. S1). For stride length, the correlation between the predicted (1.81 (*SD* 0.19) m) and calculated transition stride length (1.81 (*SD* 0.11) m) resulted in an *R* = 0.64 (*P* < 0.001)(see Supplementary Fig. S1). The difference between the two transition stride lengths was 0.2% (*SD* 8.2%). A Bland-Altman plot had limits of agreement of −15.9% and 16.4% and a regression equation of y = −0.66x + 1.2, with an *R* = −0.61 (*P* < 0.001)(see Supplementary Fig. S1). For the Froude number, the correlation between the predicted (0.57, *SD* 0.14) and calculated transition Froude number (0.48, *SD* 0.06) resulted in an *R* = 0.43 (*P* = 0.028)(see Supplementary Fig. S1). The difference between the two transition Froude numbers was −20.0% (*SD* 26.7%). A Bland-Altman plot had limits of agreement of −72.4% and 32.4% and a regression equation of y = −1.13x + 0.5, with an *R* = −0.76 (*P* < 0.001)(see Supplementary Fig. S1). For comparison, the corresponding values for the analysis of stride frequency were: *R* = 0.56 (*P* = 0.003), −0.5% (*SD* 4.2%), −8.7% and 7.7%, y = 0.06x−4.8, and *R* = 0.06 (*P* = 0.771)(see Supplementary Fig. S1).

### Effect of leg length

The leg length was neither significantly correlated with the calculated transition stride frequency (two-tailed Spearman, *P* = 0.256) nor with the predicted transition stride frequency (two-tailed Spearman, *P* = 0.361). Furthermore, the leg length was not significantly correlated with the transition velocity (two-tailed Spearman, *P* = 0.447) in line with the results from another study^36^. However, this was in contrast to the results of yet another study that revealed a moderate^34^ and positive correlation^9^.

## Discussion

Likely, the reason for the walk-to-run transition is multifactorial. At the same time, it appears that some factors have stronger influence than others. The present study applied an operational approach that links experimental observations to theory^37^ in line with previous studies^33, 38^. The theories involved in the present study suggest that locomotion is both CPG-mediated^28, 39^ and influenced by behavioural attractors^10, 30^. From the perspective of a dynamical system, the transition between walking and running is considered a spontaneous shift from one attractor gait pattern to another^2^. More precisely the question is, “What triggers the shift?” It is possible that behavioural attractors in the form of freely chosen stride frequencies during unrestricted gait conditions strongly influence the spontaneous walk-to-run transition^30^. Furthermore, it is conceivable that these particular stride frequencies to a large extent represent CPG-mediated outcomes that are evolutionary consolidated and genetically predisposed^33^. Eventually, as suggested in the present article, a combination of these two theories might contribute to explain the transition. Of interest is that nearly three decades ago Thorstensson and Roberthson (1987) speculated along the same lines by stating that “The subjective feeling that a transition at a certain speed will lead to a situation that is more comfortable might be based on previous experience in combination with information from peripheral receptors and activity in the central networks controlling locomotion”^36^. If the above considerations on a relation between a CPG-mediated outcome and behavioural attractors are correct, the stride frequency may be an influential factor for the gait transition. The present results on agreement between the calculated and predicted transition stride frequencies could be interpreted to support this proposal. A comparison between Bland-Altman analyses using stride frequency and the three other computed variables (i.e., gait velocity, stride length, and Froude number) confirmed our hypothesis related to the particular relevance of stride frequency during walk-to-run transition. For example, using velocity, stride length, and Froude number in the Bland-Altman analysis resulted in (*i*) larger relative limits of agreement and (*ii*) significant correlations between the mean values of the predicted and calculated transition values and the relative differences between the predicted and the calculated transition values. The first point suggested poorer agreement between predicted and calculated walk-to-run transition using velocity, stride length or Froude number. The second point suggested a proportional bias indicating that the predicted and calculated values do not agree through the range of measurements. Both aspects are indicators of poorer agreement. Thus, the stride frequency at freely chosen walking and running is apparently the best determinant of the walk-to-run transition as compared to the variables of velocity, stride length, and Froude number.

Replacing the predicted transition stride frequency, *d*, in eq. 1 with the calculated transition stride frequency while replacing the constant of 0.5 with an indefinite (*x*), and calculating *x* for all individual participants, resulted in a mean *x* of 0.5 (*SD* 0.1). Minimum and maximum values were 0.3 and 0.7, respectively. Most likely, a part of the latter inter-individual variation compared with a constant of 0.5 reflects motor noise, which has been described in a review^40^.

Of note is that data on preferred gait as well as walk-to-run transition in ostriches were recently reported^41^. Thus, the authors reported mean values of freely chosen velocities during freely walking and running as well as the transition velocity. In addition, polynomial fits from stride frequencies vs. velocity were reported. Based on these data and equations, mean values of freely chosen stride frequencies during walking and running as well as at the walk-to-run transition could be calculated. Further, the predicted walk-to-run transition stride frequency could be determined using the approach from the present study. We performed this analysis on the data from Daley *et al*.^41^ and found similar mean relative values of predicted and calculated walk-to-run transition stride frequencies for the ostriches. The values were 0.41 and 0.44, respectively. Based on a stated maximal stride frequency of 1.95 Hz for the ostriches, the absolute predicted and calculated transition stride frequencies amounted to similar values of 48 and 51 strides min^−1^, respectively. Although there are obvious differences between an ostrich and a human, e.g. in leg anatomy, there are at the same time essential similarities among these species. Both species are vertebrates and use the legs for locomotion purpose. In that perspective, it may not be surprising that the present suggestion of a role of stride frequency for walk-to-run transition apparently can be supported by data collected on ostriches by Daley *et al*.^41^.

When considering strengths and limitations of the present study, the following should be noted. Individual data rather than mean data were considered in the present study. This provides more detailed information than when merely considering mean data. The hypothesis of the present study involved well-established motor control theories. Combined with the present data, this led to a novel interpretation of the walk-to-run transition. In previous studies, an over-ground protocol was applied, which allowed participants to accelerate freely^42, 43^. It cannot be excluded that applying another protocol in the present study would have resulted in different results. For example, it has been reported that the walk-to-run transition is affected by treadmill belt acceleration^44^. It is also possible that a step-by-step analysis could have resulted in useful details in addition to the information from the more gross analysis in the present study. This is corroborated by the fact that the duration of the transition step is longer than the duration of the step preceding the transition step^44^. An approximately 5% drop in stride frequency at the transition stride as reported by van Caekenberghe *et al*.^44^ could have resulted in an overestimation of the calculated stride frequency in the present study. Still, the latter does not preclude the interpretation of the present results. A final note is that De Smet *et al*.^42^ concluded by hypothesizing that the walk-to-run transition might be triggered by the stride frequency reaching a certain level. This suggestion is in line with the interpretation of the present results.

The present work investigated the walk-to-run transition, which is a central phenomenon within bipedal locomotion. In comparison with previous research within the field, we focussed on the stride frequency rather than the velocity at the transition. There was agreement between two different methods (a calculation-based and a novel prediction-based) for determination of the transition stride frequency. The agreement comprised practically no mean difference (i.e. −0.3 strides min^−1^) between the two transition stride frequencies as well as a significant correlation (*R* ^*2*^ = 0.316) between them. Theories on CPG-mediated behavioural attractors in the form of stride frequencies during unrestricted gait conditions (in this study, walking and running at freely chosen velocities and freely chosen stride frequencies) were considered for interpretation of the present results. In combination with the present results, these theories support the notion that stride frequency could play a noticeable role for the walk-to-run transition in humans.

## Methods

### Participants

The 19 men and 7 women who participated were characterised by the age, height, and body mass of 26.3 (*SD* 5.4) years, 1.78 (*SD* 0.08) m, and 75.2 (*SD* 10) kg, respectively. Before testing, written informed consent was obtained from all the participants. The study was approved (N-20160003) by The North Denmark Region Committee on Health Research Ethics and conformed to the standards of the Declaration of Helsinki.

### Treadmill locomotion

The protocol was composed of four parts. The first part consisted of a 10 min familiarization during which the participant was walking on a motorised WOODWAY Pro XL treadmill (WOODWAY Inc., Waukesha, WI, USA) at 1 km h^−1^ for 1 min. Subsequently, the velocity was increased by 1 km h^−1^ each min finishing at 10 km h^−1^. This part was completed by 10 min of rest, to avoid fatigue. During the rest period, height and body mass were measured. In addition, the leg length was measured from the top of the anterior superior iliac spine to the bottom of the lateral malleolus^33^. The second part of the protocol constituted the main part. It was initiated by a bout of locomotion commenced at 3 km h^−1^ at which the participant walked for 30 s. Subsequently, the experimenters increased the velocity by 0.5 km h^−1^ every 30 s finishing with 30 s at 10 km h^−1^. In advance, the participant had been instructed to shift from walking to running whenever it felt natural during the bout. With respect to the data analysis, the first velocity at which the participant chose to run was considered the individual transition velocity. The justification for this particular procedure as well as for applying a discrete treadmill protocol was that it was consistent with many previous studies^1, 9, 12^ and thus allowed us to compare the present results with previously published findings. The second part was completed by 5 min of rest. In the third and fourth part of the protocol, the participant performed bouts of walking and running at freely chosen velocity and freely chosen stride frequencies. These two bouts occurred in counterbalanced order and were separated by 5 min of rest. In order to standardize the procedure, all participants received the same instructions. The instruction for the walking bout was as follows, “*You are now supposed to walk in a preferred and comfortable way. For example, you could imagine yourself walking on the road without any particular purpose*”. The instruction for the running bout was as follows, “*You are now supposed to run in a preferred and comfortable way. For example, you could imagine yourself running on the road without any particular purpose”*. The participant was instructed to adjust the treadmill velocity according to his or her preference and was blinded to the velocity. The bouts lasted until the participant had chosen the treadmill velocity; however, maximally 5 min and in addition 30 s for data recording. The participants wore running shoes and comfortable clothes for running.

### Stride frequencies, stride length, and Froude number

All treadmill locomotion was filmed in the sagittal plane by a silver edition of the GoPro Hero 4 (GoPro Inc., San Mateo, CA, USA). The first and last 5 s of each data recording sequence were disregarded in the analysis which was performed by VLC version 2.1.2 (VideoLAN organization, Paris, France). The stride frequency was calculated by counting the number of strides to the nearest quarter of a stride in the 20-s sequence and expressed as strides min^−1^. Stride length was calculated as velocity (in m s^−1^) divided by stride frequency (in strides s^−1^). Froude number was calculated as: Froude number = v^2^/gL, where v is velocity in m s^−1^, g is gravitational acceleration in m s^−2^, and L is leg length in m^45–47^.

### Walk-to-run transition stride frequency

Two different methods were applied for determination of the transition stride frequency. One method calculated an individual transition stride frequency based on the individual transition velocity. Initially, the stride frequency during walking at pre-set velocities was plotted as a function of the velocity for each participant (see Fig. 2 for a data example). Then, linear regression (Excel 2011, Microsoft Corporation, Bellevue, WA, USA) was applied to the plotted data, and the regression equation was used to calculate the stride frequency at the transition velocity (see Fig. 2). The latter was done since the protocol did not provide data for walking at the actual transition velocity at which running per definition was performed. For comparison, a dissimilar novel method was used to predict an individual transition stride frequency. This method was based on the theories and justification described at the beginning of the article (Fig. 1) and predicted the transition stride frequency (*d*) from the following equation:

1 $$d=0.5\times (b-a)+a$$

*a* denotes the freely chosen stride frequency (in strides min^−1^) during unrestricted walking at freely chosen velocity. *b* denotes the freely chosen stride frequency (in strides min^−1^) during unrestricted running at freely chosen velocity. For additional explanation, the reader is referred to Fig. 1.

## Electronic supplementary material

Supplementary Figure S1
