# Supplementary material for: The role of stride frequency for walk-to-run transition in humans
Source: Sci Rep. 2017 May 17;7:2010. doi: 10.1038/s41598-017-01972-1 (PMC5435734; doi:10.1038/s41598-017-01972-1)
Supplement: Supplementary file 1 — Supplementary Figure S1 [file 41598_2017_1972_MOESM1_ESM.pdf]

The role of stride frequency for walk-to-run transition in humans

Ernst Albin Hansen, Lasse Andreas Risgaard Kristensen, Andreas Møller Nielsen, Michael Voigt, Pascal Madeleine

Research Interest Group of Physical Activity and Human Performance, SMI, Department of Health Science and Technology, Aalborg University, Fredrik Bajers Vej 7D, 9220 Aalborg, Denmark.

Corresponding author:
Ernst Albin Hansen, MS, PhD, DSc
Associate Professor
Research Interest Group of Physical Activity and Human Performance, SMI,
Department of Health Science and Technology, Aalborg University
Fredrik Bajers Vej 7D, DK-9220 Aalborg, Denmark
E-mail: eah@hst.aau.dk

Supplementary figure

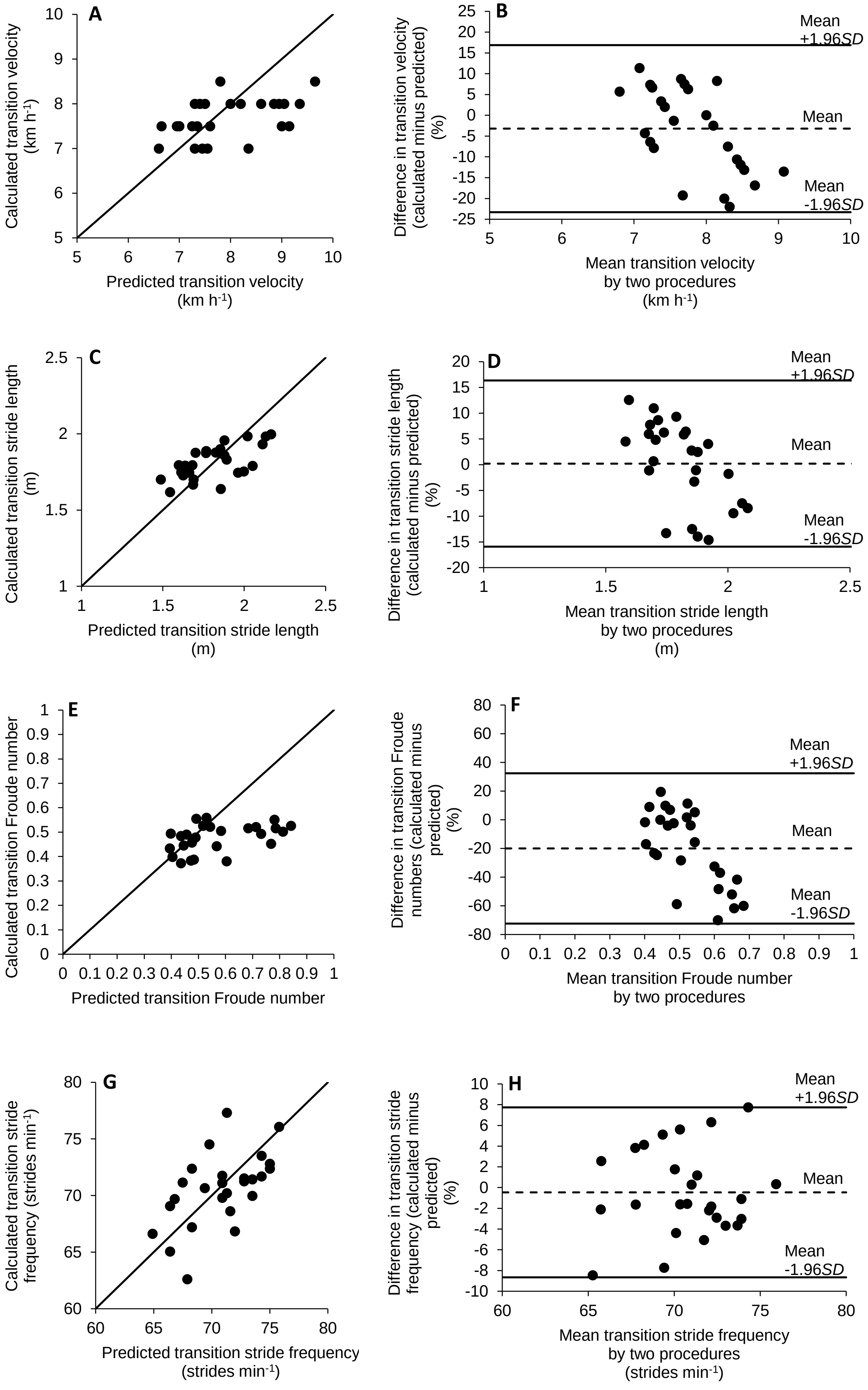

Supplementary Figure S1. A, C, E, and **G)** Calculated vs. predicted values of velocity, stride length, Froude number, and stride frequency at walk-to-run transition. *n* = 26. Lines of equality are superimposed. *R*-values are reported in the article. **B, D, F,** and **H)** Bland-Altman plots. *n* = 26. The difference between the two applied methods (predicted and calculated) for determination of a given variable at the transition stride frequency is depicted as a function of the mean value by the two methods. Regression equations as well as *R*-values are reported in the article.
